# Wearable Wellness Technology Usage and Concerns Across Middle Age and Older Adulthood

**DOI:** 10.1093/geroni/igaf122.2816

**Published:** 2025-12-31

**Authors:** Laurel Mertz, Jennifer Smith

**Affiliations:** Mather Institute, Evanston, Illinois, United States; Mather Institute, Evanston, Illinois, United States

## Abstract

Wearable technologies may enhance wellness by supporting diagnostics, self-monitoring, and behavior change. Although middle-aged to older adults may particularly benefit from wearable devices, their usage remains underexplored and may be impacted by privacy concerns, technological barriers, and affordability. This study aimed to evaluate how and why different age cohorts (adults in their 40s, 50s, 60s, and 70s) use various wearable devices and to assess their potential concerns. A total of 5,037 adults, ages 44 to 78, were recruited via an online research panel (mean age = 59.9 years, 51.7% female, 65.5% White). Participants completed a survey assessing their health monitoring using wearable technologies. Those who used wearables reported the metrics they tracked, while non-users provided reasons for their non-use. Chi-square analyses revealed that adults in their 40s used smartwatches, smart rings, and other wearables to monitor their health more than any other group. Among wearable technology users, similar proportions across age cohorts tracked blood pressure, heart rate, physical activity, sleep, and weight. However, age differences emerged in the tracking of habits, mood, screen time, and food/water intake. Common reasons for non-use across age groups included data privacy concerns, usage difficulties, cost, and lack of interest. Generally, older cohorts were more likely to report tracking their health without using wellness technologies. These results indicate that age cohorts may use wearables for different purposes, but non-users share similar concerns regardless of age. Further research is needed to explore alternative health tracking methods among older adults, and to address concerns about wearable devices.

